# Refined trimming of orbital fat in transconjunctival lower blepharoplasty: A retrospective cohort study on clinical outcomes and aesthetic assessment

**DOI:** 10.1097/MD.0000000000045314

**Published:** 2025-10-24

**Authors:** Wenbo Li, Ya Gao, Ziyi Qi, Zhongxing Li, Fenglian Wu

**Affiliations:** a Hebei Medical University, Shijiazhuang City, Hebei Province, People’s Republic of China; b Department of Surgery, Plastic and Cosmetic Surgery, First Hospital of Qinhuangdao, Qinhuangdao City, Hebei Province, People’s Republic of China.

**Keywords:** blepharoplasty, orbital fat, patient satisfaction, retrospective studies, tear trough deformity, transconjunctival

## Abstract

Herniated orbital fat and tear trough deformity are key manifestations of periorbital aging. While transconjunctival lower blepharoplasty effectively addresses these concerns, contemporary fat-preserving techniques can lead to midfacial overfilling when orbital fat volume is excessive. This study evaluates a surgical technique incorporating precise orbital fat trimming to prevent overcorrection and achieve optimal aesthetic outcomes. A retrospective cohort study was conducted on 97 patients who underwent transconjunctival lower blepharoplasty with refined orbital fat trimming between July 2019 and July 2023. All procedures were performed by a single surgeon. Outcome measures included operative time, postoperative complications, patient-reported satisfaction using the validated FACE-Q Satisfaction with Eyes scale (scored 1–4), Barton grade improvement, and objective aesthetic assessment by 2 independent blinded surgeons using a novel 5-point Likert scale evaluating 4 domains: orbital fat contour, lid-cheek junction, overall improvement, and symmetry. The mean age was 37.73 ± 4.88 years with mean follow-up of 8.2 ± 2.1 months. Mean operative time was 56.34 ± 5.61 minutes. No major complications occurred. Minor complications included transient lower eyelid swelling (100%, resolving within 8 weeks), mild chemosis (2.1%, resolving within 2 weeks), mid-facial skin numbness (3.1%, resolving within 3 weeks), and mild bleeding (7.2%, ≤0.5 mL). Patient satisfaction was high (mean FACE-Q score 3.63 ± 0.52), with 67.01% “very satisfied”, and 29.90% “somewhat satisfied.” Objective aesthetic assessment showed significant improvement (mean score 4.2 ± 0.6) with excellent inter-rater reliability (intraclass correlation coefficient = 0.89). All cases demonstrated resolution of fat herniation and significant tear trough improvement without overfilling. Refined trimming of orbital fat in transconjunctival lower blepharoplasty effectively addresses both fat herniation and tear trough deformity while preventing midfacial overfilling. This technique represents an advance in personalized lower blepharoplasty, demonstrating high safety, efficacy, and patient satisfaction through standardized outcome measures.

## 1. Introduction

Herniated orbital fat in the lower eyelid and tear trough deformity are important signs of orbital and mid-face aging, and are also popular issues in cosmetic surgery research and resolution. Periorbital aging is a complex process characterized by multifaceted anatomical changes. The attenuation of the orbital septum leads to anterior pseudoherniation of the intraorbital fat pads, creating the typical bulging of the lower eyelid. Concurrently, age-related resorption of the sub-orbicularis oculi fat (SOOF) and deep medial cheek fat, combined with descent of the malar fat pad, contributes to the formation of the tear trough deformity and mid-cheek groove. This results in a prominent lid-cheek junction, casting shadows that accentuate an aged and tired appearance.^[1]^ The primary surgical challenge, therefore, lies in addressing both herniation of orbital fat and volume deficiency in the tear trough region to restore a smooth and youthful transition.

The transconjunctival approach has become a mainstream technique for lower eyelid rejuvenation. It is specifically indicated for patients with moderate to severe orbital fat pseudoherniation (typically Barton grade II–III) and a visible tear trough deformity, but with good lower eyelid tone, minimal to no skin excess, and no significant eyelid laxity or malar festoons. Its major advantages include the absence of a visible external scar, preservation of the orbicularis oculi muscle, and a reduced risk of eyelid malposition compared to transcutaneous approaches.

In the early stage, simple orbital fat removal was mainly used for correction, but it could not solve the depression of the tear trough deformity, and might lead to the depression of the lower eyelid. Since then, Trepsat^[2]^ proposed that backfilling the removed free orbital fat to the tear trough can improve the depression of the tear trough deformity to a certain extent, but the backfilled fat will be partially absorbed,^[3,4]^ and the effect is unstable. Goldberg^[5]^ proposed a transconjunctival approach to release orbital fat to fill the tear trough, which further improved the correction effect of tear trough deformity, but there may be residual or recurrent problems after surgery. The “S.O.F.T.” method^[6]^ proposed by Jin not only solved the problem of orbital fat protrusion, but also significantly improved the aesthetics of tear trough deformity, and it greatly reduces the operation difficulty of plastic surgeons.

However, a common limitation persists across these advanced fat-preserving techniques: when the volume of the orbital septal fat is excessive, its transposition can lead to overfilling of the mid-face, resulting in an unnatural convexity. This issue is particularly relevant in patients with robust orbital fat pads. In this study, we employed a surgical technique that incorporates precise, intraoperative trimming of the orbital fat pedicle, aiming to address this specific challenge of excess volume during transconjunctival lower blepharoplasty and to achieve a more customized and natural aesthetic outcome.

## 2. Methods

This is a retrospective study of 357 patients who underwent transconjunctival lower blepharoplasty between July 2019 and July 2023. Of these, 97 cases were identified with excess orbital fat volume during the operation, which were the focus of this research. The study was approved by the ethics committee of the First Hospital of Qinhuangdao and adhered to the guidelines of the Declaration of Helsinki.

All operations were performed by the same surgeon (F.W.), and informed consent was obtained from each patient. Patient inclusion criteria: herniated orbital fat in the lower eyelid, tear trough deformity, a certain degree of mid-cheek depression, excess orbital fat volume during the operation. Exclusion criteria: lower eyelid skin and lower eyelid laxity, history of lower eyelid and/or mid-face surgery, injections of fillers or fat into the lower eyelid and/or mid-face, history of trauma in the lower eyelid and/or mid-face, suffering from other diseases such as facial nerve paralysis, ocular inflammation, significant upper eyelid ptosis requiring surgical correction.

It is important to note that this study specifically focuses on patients amenable to the transconjunctival approach. Consequently, patients who presented with indications warranting an external incision (such as significant dermatochalasis [excess skin], prominent malar festoons, or severe lower eyelid laxity requiring canthopexy/canthoplasty) were not included in this cohort and underwent a transcutaneous lower blepharoplasty instead.

Consult the medical records to obtain and record the patient’s age, operation time (minutes), postoperative complications (e.g., lower eyelid swelling, chemosis, bleeding, lower eyelid infection, numbness of mid-face skin, residual or recurrent orbital fat, binocular diplopia, lower eyelid ectropion). Patient-reported satisfaction was assessed using the validated FACE-Q Eye Module, specifically the “Satisfaction with Eyes” scale (scored from 1 = very dissatisfied to 4 = very satisfied) at the 6-month postoperative follow-up.^[7]^ The Barton grade^[8]^ at the 6-month postoperative follow-up were compared with those of the preoperative assessment. Aesthetic outcomes were assessed by 2 independent, blinded plastic surgeons based on standardized preoperative and 6-month postoperative photographs. Evaluation was performed using a novel 5-point Likert scale specifically designed for this study, which rated 4 distinct domains: Orbital Fat Contour: (1 = Severe bulging, 5 = Optimal contour); Lid-Cheek Junction: (1 = Severe step-off, 5 = Smooth transition); Overall Aesthetic Improvement: (1 = Worse, 5 = Very much improved); Symmetry: (1 = Severe asymmetry, 5 = Excellent symmetry). The overall aesthetic score for each patient was calculated as the mean of the scores from the 2 surgeons across all 4 domains. Inter-rater reliability between the 2 surgeons was assessed using the intraclass correlation coefficient (ICC) for the overall aesthetic scores. An ICC value >0.75 was considered to represent excellent agreement.

### 2.1. Surgical technique

**Figure 1.**
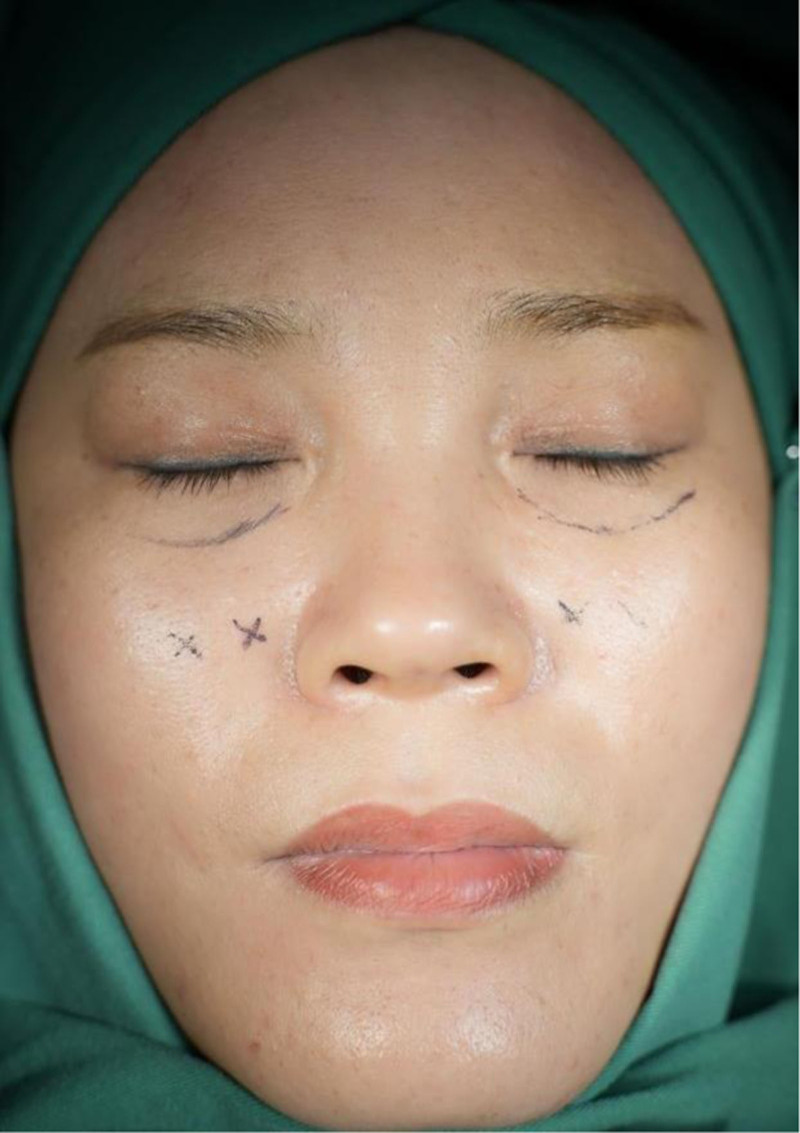
*Preoperative planning*: mark the herniated orbital fat in the lower eyelid and the extent of the tear trough deformity. Also, mark the approximate location for external fixation.

*Preoperative planning*: The patient is to sit or stand with eyes looking straight ahead. Identify and mark the herniated orbital fat in the lower eyelid and the extent of the tear trough deformity. Also, mark the approximate location for external fixation (Fig. 1).

*Operation process*: All operations were performed under local anesthesia with the preparation of 2% lidocaine mixed at a ratio of 1:200,000 with epinephrine. The infraorbital nerve and zygomaticofacial nerve were anesthetized through the injection of 2 mL of the aforementioned lidocaine solution, aimed at numbing the middle cheek area. Local infiltration anesthesia was administered through the conjunctiva of the lower eyelid.

**Figure 2.**
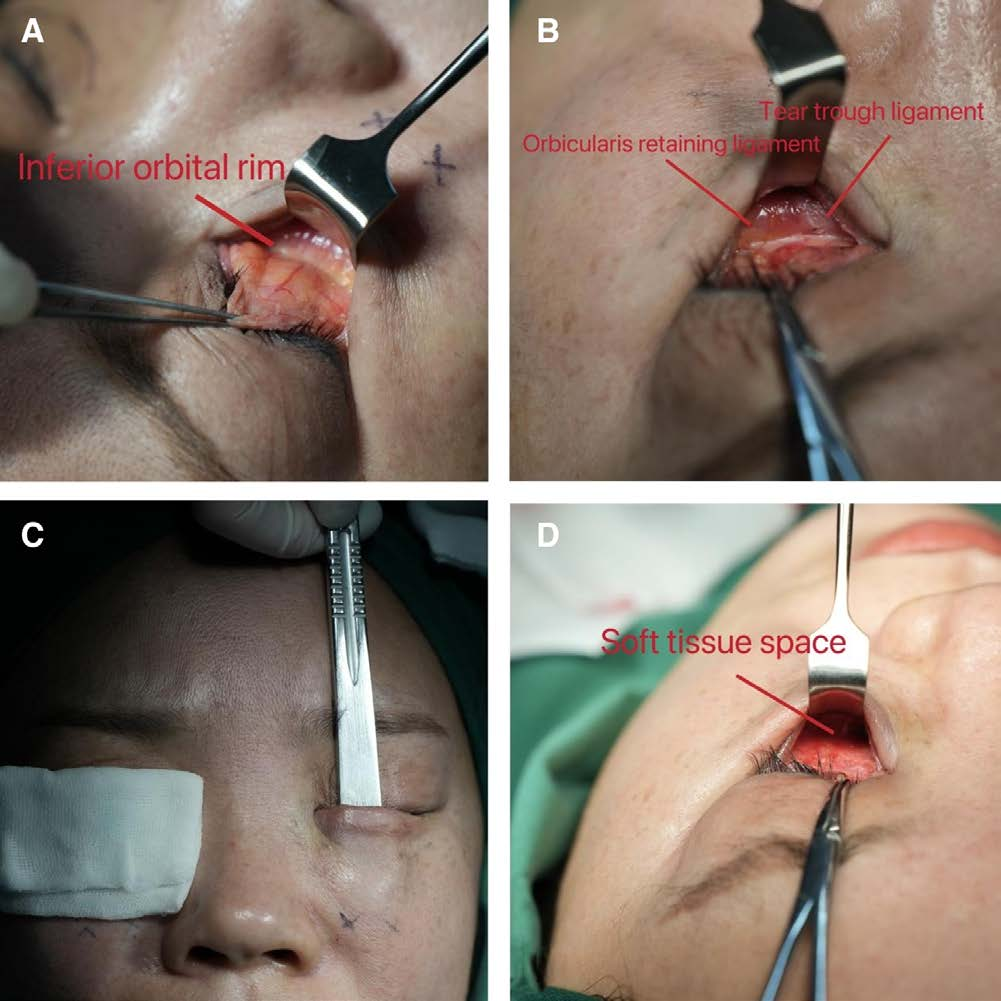
*Operation process*: soft tissue space preparation. (A) Dissect along the preseptal plane of the orbital septum until you reach the arcus marginalis of the inferior orbital rim. (B) The orbicularis retaining ligament and tear trough ligament were thoroughly relaxed using electrocautery. (C) Insert the handle of the scalpel into the lower eyelid surgical area and perform blunt dissection caudally to further enlarge the premaxillary space. (D) Connect the premaxillary space with the prezygomatic space to form a sufficient soft tissue space.

*Soft tissue space preparation*: An incision approximately 15 to 18 mm in length was made transconjunctivally along the horizontal direction of the lower eyelid using sharp-tipped electrocautery. Dissect along the preseptal plane of the orbital septum until you reach the arcus marginalis of the inferior orbital rim (Fig. 2A). The assistant utilized the eyelid retractor to adequately expose the lower eyelid surgical site, and the orbicularis retaining ligament and tear trough ligament were thoroughly relaxed using electrocautery (Fig. 2B).The sign of whether the tear trough ligament has been fully released is the exposure of the levator labii superioris. The visualization of the levator labii superioris indicates that the tear trough ligament has been completely released and has entered the premaxillary space. Insert the handle of the scalpel into the lower eyelid surgical area and perform blunt dissection caudally to further enlarge the premaxillary space. After complete dissection the premaxillary space, use the handle of the scalpel again to perform a lateral shift, extending to the prezygomatic space (Fig. 2C). Connect the premaxillary space with the prezygomatic space to form a sufficient soft tissue space. The dissection range: the distance from the orbital rim to the caudal boundary of the premaxillary space is 20 to 25 mm (Fig. 2D).

**Figure 3.**
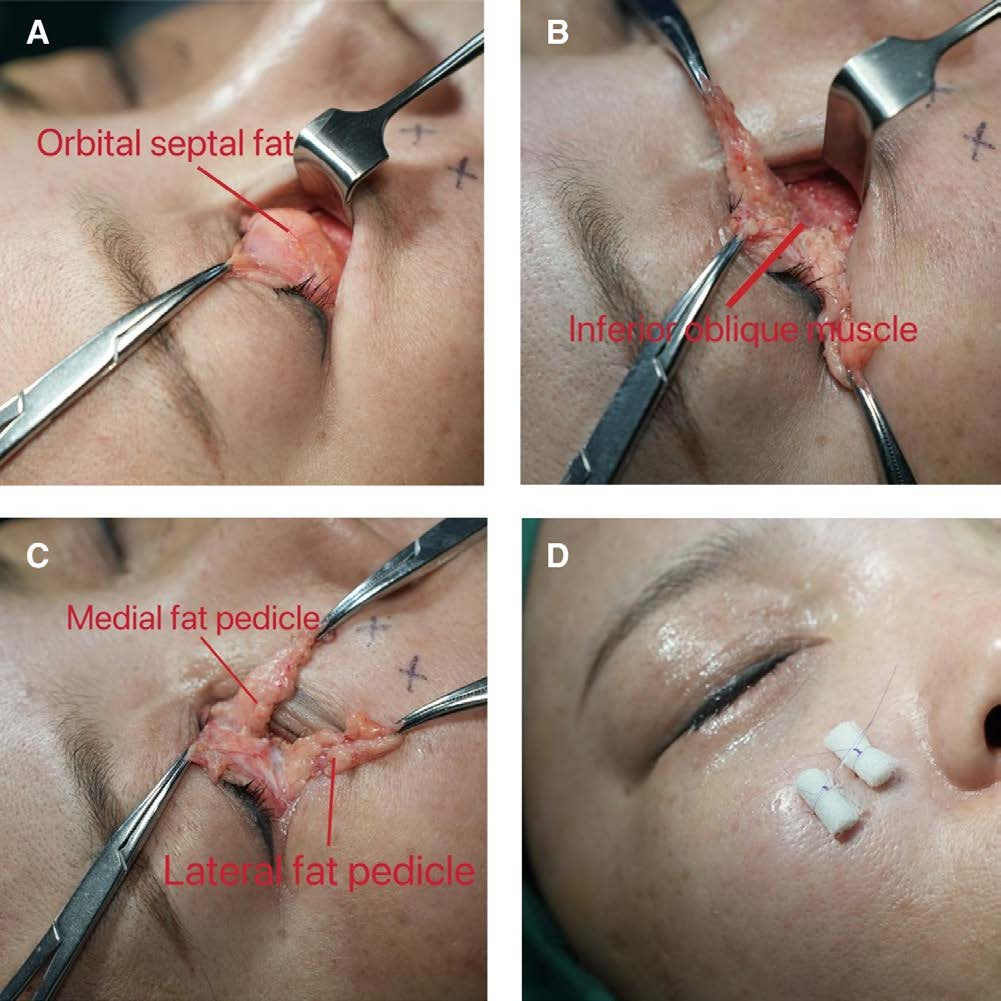
*Operation process*: orbital fat release, refined trimming of orbital fat and percutaneous external fixation of orbital fat. (A) A transverse incision is made along the orbital rim to expose the orbital septum fat. (B) Carefully stripping the fibrous connective tissue between the fat masses, especially near the inferior oblique muscle. Ensure the orbital fat is fully exposed and divide it into 2 fat masses. (C) After refined trimming. Fine-tune the orbital fat removal in small amounts multiple times until the patient’s appearance is satisfactory. (D) Percutaneous external fixation of orbital fat.

*Orbital fat release*: Using sharp-tipped electrocautery, a transverse incision is made along the orbital rim to expose the orbital septum, revealing the orbital septal fat (Fig. 3A). Dissect the arcuate expansion of Lockwood ligamen to prepare for the insertion of the pedicle fat flap into the soft tissue space. By carefully stripping the fibrous connective tissue between the fat masses, especially near the inferior oblique muscle, the membranous structure that encapsulates the orbital fat is released, allowing the orbital fat to be fully exposed. The exposed orbital fat is divided into 2 groups of pedicle fat flaps: the medial fat pedicle (including the medial fat pad) and the lateral fat pedicle (including the central orbital fat pad and lateral orbital fat pad). The criterion for complete release is the absence of significant spontaneous retraction of the pedicle fat flap when it is left in a relaxed state (Fig. 3B).

*Refined trimming of orbital fat*: After releasing the orbital fat, the surgeon evaluates whether the fat pedicle needs to be trimmed based on its size and severity of the tear trough deformity. Perform precise and individualized trimming on the sagittal and coronal planes of the fat pedicle using sharp-tipped electrocautery. A small amount of trimming is initially carried out, with a width of approximately 1 to 1.5 mm, and the fat pedicle fat is laid flat into the soft tissue space. The appearance of the patient is observed and evaluated to determine whether further trimming is necessary. If further trimming is required, the above steps are repeated. This process is repeated until the surgeon is satisfied with the patient’s appearance (Fig. 3C).

*Percutaneous external fixation of orbital fat*: The assistant apply gentle upward traction of the midcheek with the eyelid retractor, fully exposing the soft tissue space to allow the passage and externalization of the sutures. The surgeon sutures the distal end of the medial fat pedicle with 6-0 polydioxanone (PDSTM II; Ethicon, Inc, Cincinnati), and then externalizes it through the skin at the lower boundary of the soft tissue space. After the suture is passed out of the skin, a small cylinder prepared using vaseline gauze is placed on the skin surface and knotted. The lateral fat pedicle is fixed in the same manner. Then, fix the other side in the same manner (Fig. 3D). Replace the orbital septum and conjunctiva, and do not suture the wound. Squeeze erythromycin eye ointment into the conjunctiva, and apply appropriate pressure and bandaging.

*Postoperative care*: Apply cold compresses intermittently to the surgical area for 48 hours post-operation. Remove external sutures after 1 week, avoid massaging the eyelids for 2 weeks, and refrain from wearing contact lenses for 4 weeks.

### 2.2. Statistical analysis

Statistical analyses were performed using SPSS software (version 26.0; IBM Corp., Armonk). Descriptive data are presented as mean ± standard deviation for continuous variables and as number (percentage) for categorical variables. To investigate factors influencing patient satisfaction, univariate analyses were conducted. The Pearson correlation coefficient was used to assess the relationship between the FACE-Q satisfaction score (continuous variable) and continuous variables (age, body mass index [BMI], operative time). The Spearman rank correlation coefficient was used for the ordinal variable (preoperative Barton grade). A *P*-value of <.05 was considered statistically significant.

## 3. Results

The average age of all 97 patients is 37.73 ± 4.88 years (ranging, 27–46 years). Among them, there are 15 males and 82 females. The average operating time was 56.34 ± 5.61 minutes (range, 45–82 minutes).The mean follow-up time was 8.2 ± 2.1 months (range, 6–14 months). A subset of 23 patients (23.7%) underwent simultaneous upper blepharoplasty. A subgroup analysis confirmed that the concurrent performance of an upper blepharoplasty had no significant impact on the outcomes (satisfaction rates, complication rates) of the lower blepharoplasty procedure documented in this study. Throughout the follow-up period, no patient required reoperation, revisional procedures, or adjunctive fat grafting to the periorbital region.

**Figure 4.**
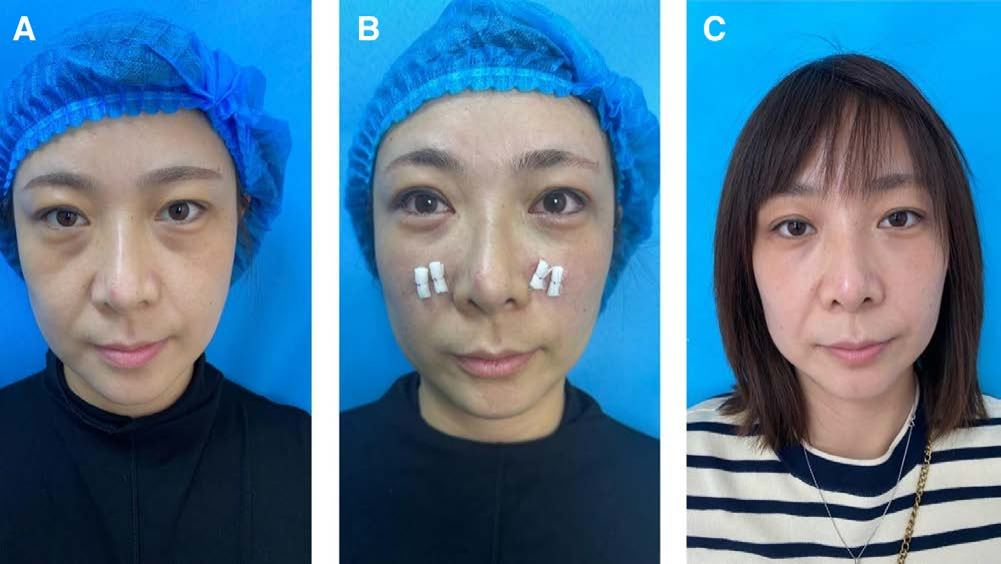
Photos of a female patient showing early results of refined trimming of orbital fat in transconjunctival lower blepharoplasty. (A) This is a 36-year-old woman with herniated orbital fat in the lower eyelid and tear trough deformity. (B) There is some lower eyelid edema immediately after the surgery. (C) At 1 months after the surgery, the protrusion of orbital fat was resolved, and the deformity of the tear trough also showed significant aesthetic improvement without the issue of excessive filling.

**Figure 5.**
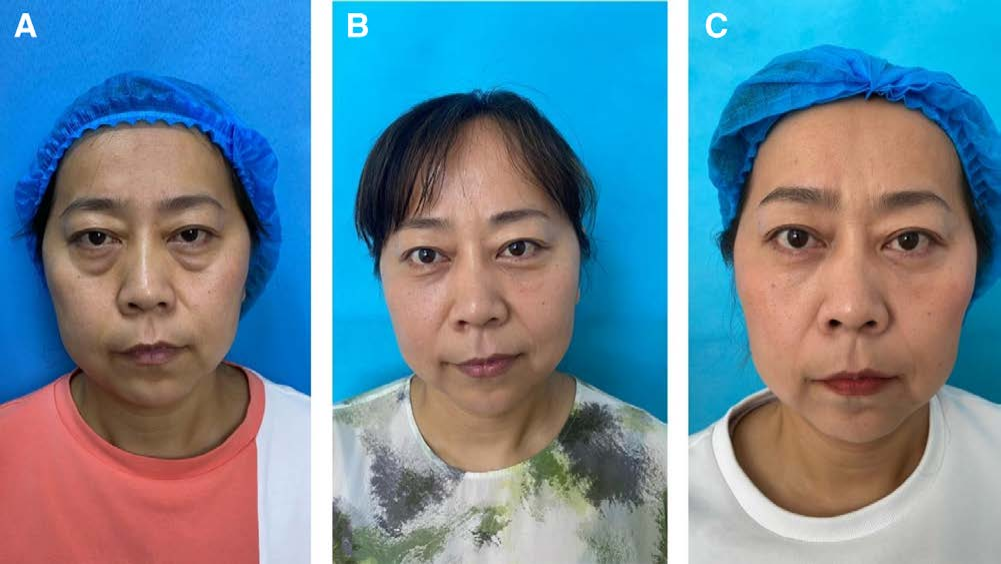
Photos of a female patient showing long-term results of refined trimming of orbital fat in transconjunctival lower blepharoplasty. (A) This is a 45-year-old woman with Herniated orbital fat in the lower eyelid and tear trough deformity. (B) She is shown 3 months after the surgery. (C) She is shown 6 months after the surgery, the protrusion of orbital fat was resolved, and the deformity of the tear trough also showed significant aesthetic improvement without the issue of excessive filling.

Patient-reported outcomes, as measured by the FACE-Q Satisfaction with Eyes scale, demonstrated high levels of satisfaction. The mean satisfaction score was 3.63 ± 0.52 (range 1–4). Of the 97 patients, 65 (67.01%) were “very satisfied” (score of 4), 29 (29.90%) were “somewhat satisfied” (score of 3), 3 (3.09%) were “somewhat dissatisfied” (score of 2), and 0 (0%) were ``very dissatisfied’’ (score of 1). The sub-group analysis confirmed that simultaneous upper blepharoplasty did not significantly affect the FACE-Q satisfaction scores (*P* > .05). At the final follow-up, 1 case was assessed as Barton grade II, and 2 cases as Barton grade I. The objective aesthetic assessment by blinded surgeons yielded high scores. The mean overall aesthetic score was 4.2 ± 0.6 (range 1–5), indicating significant improvement. The inter-rater reliability was excellent, with an ICC of 0.89 for the overall aesthetic scores, confirming a high level of agreement between the 2 independent assessors. Immediately after surgery, the problem of herniated orbital fat was resolved, and the tear trough deformity also underwent significant aesthetic improvement without the occurrence of excessive filling (Figs. 4 and 5). Univariate analysis was performed to identify factors correlating with patient-reported satisfaction (FACE-Q score). No significant correlations were found between the FACE-Q satisfaction score and patient age (*R* = 0.12, *P* = .252), BMI (*R* = 0.08, *P* = .442), or operative time (*r* = −0.09, *P* = .391). Furthermore, there was no significant correlation between preoperative Barton grade and postoperative satisfaction (*ρ* = 0.10, *P* = .336).

*Complications*: Swelling of the lower eyelid began to appear 24 to 48 hours after surgery, which is the most common postoperative complication for all patients. The swelling began to subside after 72 hours, none lasted beyond 8 weeks. After surgery, 2 cases exhibited mild chemosis, which resolved spontaneously within 2 weeks; 3 cases exhibited mid-facial skin numbness, which resolved spontaneously within 3 weeks. Mild oozing of blood occurred in 7 patients within the first week after surgery, with no >0.5 mL per patient. No other postoperative complications (for example lower eyelid infection, residual or recurrent orbital fat, binocular diplopia, lower eyelid ectropion).

## 4. Discussion

With the increasing awareness of personalized and precision, lower eyelid blepharoplasty surgery has become distinct from traditional methods, and the rejuvenation surgery for the lower eyelid is becoming increasingly precise. Personalized surgical approaches are tailored to the individual circumstances of each patient seeking aesthetic enhancement. In transconjunctival lower blepharoplasty, there exists a problem where the orbital fat volume differs among individual patients: insufficient orbital fat cannot adequately correct tear trough deformity; excessive orbital fat can lead to overfilling of the mid-face. The surgical approach for refined trimming of orbital fat lies in addressing the issue of excess orbital fat during the release of orbital septum fat through the transconjunctival approach.

Firstly, the preparation of soft tissue spaces is one of the key factors determining the surgical outcome. Selecting an appropriate transposition gap for the refined trimmed orbital fat is key to correcting the deformity of the tear trough. With the advancements in various studies, the planes that accommodate the transferred fat in transconjunctival lower blepharoplasty include the subperiosteum, supraperiosteum, and intra-SOOF.^[9,10]^ The anatomical hierarchy of the subperiosteal space is clear,^[11]^ offering advantages of high safety and clear anatomical visualization. However, due to the limited separability of the subperiosteal space compared to soft tissue spaces, it is insufficient to accommodate the transposed orbital fat. There are important structures such as nerves and blood vessels present in the supraperiosteal plane. If damage occurs inadvertently, complications such as intraoperative bleeding, postoperative bruising and swelling, and numbness in the surgical area may arise, potentially leading to a prolonged recovery period.^[12]^ During the dissection of SOOF, there are zygomatic perforating branches of the facial nerve and deep lymphatic tissues. If they are damaged, it may lead to denervation of the orbicularis oculi muscle, resulting in obvious postoperative edema and slower recovery.^[13,14]^

Mendelson et al^[15,16]^ discovered a distinct anatomical plane-the premaxillary space and prezygomatic space. As natural loose spaces in the mid-face which are situated underneath the SOOF and over the levator labii superioris muscle, these spaces are easily separated by blunt dissection. These spaces providing ample space for the transposed orbital fat after refined trimming. The orbicularis retaining ligament and tear trough ligament are considered to be the preferably released for better integration of the lower eyelid-cheek junction.^[17]^ As a soft tissue space, it is rich in blood supply, ensuring the survival of the transposed orbital fat after precisely trimmed. Moreover, there are no major blood vessels distributed within the space, and there is no zygomatic branch of the facial nerve or deep lymphatic tissue.^[18]^

Currently, the fixation methods for orbital fat transposition primarily fall into 2 categories: internal fixation and percutaneous external fixation. Internal fixation is relatively stable, but due to the limited operational space, it poses operational challenges and is time-consuming and laborious. In this study, after sutures were passed through the skin, small cylinders made of vaseline gauze were placed on the skin surface and knotted. This method is quick and easy, avoiding the possibility of leaving pinpoint scars due to overly tight knotting. None of the cases in this study exhibited skin scars or pigmentation, indicating the effectiveness of this fixation method. The release method employed in this study involves thoroughly loosening the fibrous tissue of the orbital fat and externally fixing it to the skin surface. This approach effectively reduces the likelihood of recurrence.

A critical consideration in fat repositioning techniques is the volume of the orbital fat pedicle itself. While transposition addresses tear trough depression, it can inadvertently create a new problem of overfilling or a palpable, unnatural convexity in the mid-face if the pedicle is excessively bulky. This issue of iatrogenic overcorrection is precisely what our technique of refined trimming seeks to mitigate. Our approach can be conceptualized as a middle ground between 2 established philosophies: the purely reductive approach of classic fat excision (which risks hollowing) and the purely additive approach of unmodified fat repositioning (which risks overfilling). By permitting intraoperative, incremental tailoring of the fat volume, we combine the benefits of both: we reduce herniation while simultaneously utilizing the patient’s own tissue for augmentation, but in a controlled manner that aims for an optimal aesthetic contour rather than simply maximal volume transfer. This precise, conservative excision enhances surgical safety, particularly for less experienced surgeons, by minimizing the irreversible step of over-resection.

Our study cohort, predominantly composed of East Asian patients, provides specific insights into the application of refined orbital fat trimming in this population. East Asian individuals often exhibit distinct midfacial anatomy characterized by a higher degree of midface fullness, a broader and less projecting nasal bridge, and a tendency toward a fuller lateral cheek contour. These characteristics, rooted in population-specific genetic variations that influence craniofacial development,^[19]^ present both an opportunity and a challenge for lower blepharoplasty. The opportunity lies in the often ample volume of orbital fat available for transposition. The challenge, however, is that the transposition of an unmodified, voluminous orbital fat pedicle in a midface that is already relatively full can easily lead to overcorrection and an unnatural, convex malar appearance (precisely the issue our technique aims to prevent).

In 357 cases of transconjunctival lower eyelid blepharoplasty, it was observed during the surgical procedure that there were significant individual differences in orbital fat volume, even among patients with the same Barton classification. Among them, 97 cases had the problem of excess volume after releasing orbital fat, including 59 cases of Barton grade III, 36 cases of Barton grade II, and 2 cases of Barton grade I. The approach adopted in this study involves personalized and refined trimming of orbital fat based on individual differences. During the trimming process, constant observation of the patient’s appearance is conducted to determine whether further trimming is necessary. Plastic surgery is gradually evolving towards a more personalized and precised direction, encompassing precise control over tissue removal and aesthetic evaluation of overall morphology. The precised orbital fat trimming technique adheres to the concept of step-by-step removal, representing a cautious and meticulous excision technique. It not only facilitates personalized local excision but also ensures enhanced surgical safety. Avoid trimming too much at once, which may lead to insufficient volume and result in inadequate correction of the deformity of the tear trough. The orbital septal fat exhibits significant interindividual variability, making it difficult to accurately predict its volume, thickness, and blood supply. Additionally, it is challenging to simulate its postoperative morphology preoperatively. Therefore, refined trimming of orbital fat largely relies on the surgeon’s experience and intraoperative judgment. For experienced senior doctors, accurate intraoperative judgment often enables one-time trimming with appropriate extent. However, for junior doctors, reducing intraoperative uncertainty and enhancing surgical safety are more crucial. Determining the appropriate amount of orbital fat retention also poses a challenge, thus giving rise to the need for precised orbital fat trimming.

The high scores observed in both the patient-reported outcomes (FACE-Q) and the objective surgeon assessment using the novel Likert scale demonstrate a strong concordance between patient perception and surgical evaluation, further validating the efficacy of the technique. Furthermore, the univariate analysis revealed no significant correlation between postoperative satisfaction and patient age, BMI, operative time, or preoperative Barton grade. This suggests that the high satisfaction rates and consistent outcomes achieved with this technique are robust across a variety of patient demographics and clinical presentations.

It is crucial to emphasize that the technique of refined orbital fat trimming described herein is not intended as a universal, “one-size-fits-all” solution for lower blepharoplasty. Rather, it occupies a specific and valuable niche in the surgical armamentarium. This approach is ideally indicated for a well-defined patient profile: those with moderate-to-severe orbital fat prolapse (typically corresponding to Barton grades II or III), who have good innate midface support and volume, and who present with minimal to no excessive skin laxity (dermatochalasis). For these patients, the transconjunctival approach combined with precise fat modulation provides an optimal solution to correct bulging and tear trough deformity while preventing midfacial overfilling.

Conversely, this technique is not suitable for patients with significant preexisting lower eyelid laxity, severe skin excess, or prominent malar festoons, as these conditions require additional procedures such as canthoplasty or skin resection via a transcutaneous approach. Similarly, patients with severe tear trough deformities coupled with profound midface volume deficiency may benefit more from a combination of fat repositioning and adjunctive autologous fat grafting to achieve adequate augmentation. Therefore, meticulous patient selection based on a thorough anatomical and aesthetic analysis remains the cornerstone of success, and this technique should be viewed as a powerful tool for a specific set of indications, not a replacement for other established methods.

This study has several limitations that should be considered. First, its retrospective design inherently carries risks of selection and information bias, despite our efforts to apply strict inclusion and exclusion criteria. Second, the lack of a control group (e.g., patients treated with standard fat repositioning without trimming) prevents definitive comparative conclusions regarding the superiority of the refined trimming technique; the favorable outcomes reported herein should be interpreted as preliminary and associative. Third, although we have now implemented the validated FACE-Q instrument in our analysis, initial patient satisfaction was assessed using a non-validated, self-reported scale, which may limit the robustness of our early satisfaction data. Finally, while the aesthetic assessment by 2 blinded surgeons demonstrated excellent inter-rater reliability (ICC = 0.89), the potential for observer bias cannot be completely eliminated, and the novel rating scale, although structured, has not itself been externally validated. Future prospective, randomized controlled studies comparing this technique to existing standards, with long-term follow-up and fully validated outcome measures, are warranted to confirm our findings.

## 5. Conclusions

The refined orbital fat trimming technique presented in this study provides an effective and safe solution for addressing both orbital fat pseudoherniation and tear trough deformity while preventing midfacial overfilling (a recognized limitation of conventional fat repositioning techniques). Our results demonstrate that this approach enables precise, individualized contouring of the lower eyelid–cheek junction, leading to significant aesthetic improvement and high patient satisfaction as measured by validated outcome instruments. The procedure’s efficacy is further supported by objective assessment using a standardized evaluation scale, which showed excellent inter-rater reliability.

This technique expands the surgical armamentarium for lower blepharoplasty by offering a tailored approach that occupies an important niche between traditional fat excision and unmodified fat transposition. It is particularly valuable for patients with robust orbital fat pads and those seeking natural-looking results without excessive midfacial fullness. While our findings are promising, further prospective comparative studies with long-term follow-up are warranted to confirm the advantages of this technique over existing methods.

Ultimately, refined orbital fat trimming represents an advancement in personalized lower eyelid rejuvenation, aligning with modern trends toward precision surgery that prioritizes individualized patient anatomy and aesthetic goals.

## Author contributions

**Conceptualization:** Wenbo Li, Zhongxing Li, Fenglian Wu.

**Data curation:** Wenbo Li, Ya Gao, Ziyi Qi.

**Formal analysis:** Fenglian Wu.

**Investigation:** Ya Gao.

**Methodology:** Wenbo Li, Fenglian Wu.

**Project administration:** Ya Gao, Ziyi Qi.

**Resources:** Ya Gao.

**Supervision:** Wenbo Li, Ziyi Qi, Zhongxing Li, Fenglian Wu.

**Validation:** Ziyi Qi.

**Visualization:** Zhongxing Li.

**Writing – original draft:** Wenbo Li, Ya Gao.

**Writing – review & editing:** Zhongxing Li, Fenglian Wu.
